# Dietary supplementation with either saturated or unsaturated fatty acids does not affect the mechanoenergetics of the isolated rat heart

**DOI:** 10.1002/phy2.272

**Published:** 2014-03-24

**Authors:** Soyeon Goo, June‐Chiew Han, Linley A. Nisbet, Ian J. LeGrice, Andrew J. Taberner, Denis S. Loiselle

**Affiliations:** 1 Auckland Bioengineering Institute, The University of Auckland, Auckland, New Zealand; 2 Department of Physiology, The University of Auckland, Auckland, New Zealand; 3 Department of Engineering Science, The University of Auckland, Auckland, New Zealand

**Keywords:** Cardiac efficiency, fish oils, working‐heart preparation

## Abstract

It is generally recognized that increased consumption of polyunsaturated fatty acids, fish oil (FO) in particular, is beneficial to cardiac and cardiovascular health, whereas equivalent consumption of saturated fats is deleterious. In this study, we explore this divergence, adopting a limited purview: The effect of dietary fatty acids on the mechanoenergetics of the isolated heart per se. Mechanical indices of interest include left‐ventricular (LV) developed pressure, stroke work, cardiac output, coronary perfusion, and LV power. The principal energetic index is whole‐heart oxygen consumption, which we subdivide into its active and basal moieties. The primary mechanoenergetic index of interest is cardiac efficiency, the ratio of work performance to metabolic energy expenditure. Wistar rats were divided into three Diet groups and fed, ad libitum, reference (REF), fish oil‐supplemented (FO), or saturated fatty acid‐supplemented (SFA) food for 6 weeks. At the end of the dietary period, hearts were excised, mounted in a working‐heart rig, and their mechanoenergetic performance quantified over a range of preloads and afterloads. Analyses of Variance revealed no difference in any of the individual mechanoenergetic indices among the three Diet groups. In particular, we found no effect of prior dietary supplementation with either saturated or unsaturated fatty acids on the global efficiency of the heart.

## Abstract (precis)

Literature reports have claimed profound effects of dietary supplementation with either saturated or polyunsaturated fatty acids on the contractile efficiency of the heart – diminishing and enhancing efficiency, respectively. We have mimicked the experimental protocols used in those reports and find no effect of diet on any index of cardiac mechanoenergetics, including efficiency.

## Introduction

The “diet‐heart” hypothesis (Erkkilä et al. 2008) states that a diet high in saturated fatty acids (SFAs) is harmful to the heart, whereas a diet rich in polyunsaturated fatty acids (PUFA), especially n‐3 fish oils (FO), is beneficial. Although the concept is not without controversy (Ravnskov 1998; Siri‐Tarino et al. 2010), a body of evidence from interventional investigations (Mustad et al. 1997), as well as both clinical trials and epidemiological studies (Oh et al. 2005; Mozaffarian et al. 2010; Siri‐Tarino et al. 2010; Acherjee et al. 2013), suggests that SFA‐rich diets increase the risks of coronary vascular disease. It is probably because of the specific focus on coronary vascular disease that there has been scant attention given to the direct effects of SFAs on myocardial tissue per se. We are aware of only a modest number of relevant studies (de Deckere and ten Hoor 1979; De Deckere and Ten Hoor 1980; Charnock et al. 1987; Hartog et al. 1987; Karmazyn et al. 1987; Demaison et al. 2000; Pepe and McLennan 2002, 2007; Billman et al. 2010; McLennan et al. 2012) chief among which are those of Pepe and McLennan (2002, 2007) who showed a halving of the contractile efficiency of hearts isolated from animals on a high‐SFA diet *vis‐à‐vis* those on a diet of standard rat chow.

The beneficial effects of a diet rich in unsaturated fats, particularly fish oils (FO), are many (Mente et al. 2009; Fares et al. 2014) and largely accepted (Kromhout et al. 2012). Purported benefits include reduction in heart rate (Grimsgaard et al. 1998; Peoples et al. 2008; Kang 2012), increase in heart rate variability (Sauder et al. 2013; Xin et al. 2013), and a reduction in the incidence of life‐threatening cardiac arrhythmias (Billman et al. 1994; Kang et al. 1995; Pepe and McLennan 1996; Negretti et al. 2000; Leaf 2006; Nodari et al. 2011). Once again, the literature aimed at revealing the mechanoenergetic consequences of a FO‐rich diet to myocardial tissue per se is sparse. In particular, little attention has been given to the quantification of the effect on myocardial contractile efficiency. Once again, the especially germane publications are those of Pepe & McLennan who have reported a doubling (Pepe and McLennan 2002) and fourfold (Pepe and McLennan 2007) increase in cardiac efficiency of hearts isolated from animals on a high‐FO diet.

The experience of our group in measuring muscle efficiency, whether cardiac or skeletal (Smith et al. 2005), across a range of species (Loiselle and Gibbs 1977), while utilizing both whole hearts (Goo et al. 2013; Han et al. 2014) and isolated, multicellular preparations (Han et al. 2012, 2013), together with measurement instrumentation including flat‐bed thermopiles (Loiselle 1979) and a flow‐through microcalorimeter (Taberner et al. 2011; Han et al. 2013, 2014), has left us skeptical of results showing either a halving, doubling, or quadrupling of total efficiency of the nonpathological heart – arising from *any* intervention. It is this skepticism that has provided the principal motivation for our investigation. Bolstering our skepticism is our difficulty in conceiving of the parallel changes that would have to occur in the mitochondria of healthy animals to underwrite changes in cardiac efficiency of the extents reported.

## Material and Methods

### Preparation of animal and diets

Experiments were conducted in accordance with protocols approved by The University of Auckland Animal Ethics Committee (Approval R787).

Male Wistar rats (*N* = 36), aged 6 weeks to 7 weeks and weighing 250 g to 350 g, were randomly assigned to one of the three different “Diet” regimens: reference (REF), fish oil rich (FO), and saturated fatty acid (SFA) rich. Food was prepared such that each diet had a unique fatty acid composition. Different amounts of FO and beef fat (as the source of SFAs) were added to fat‐free rat pellets (TD.033143, 62% Sucrose Diet (No Fat), Harlan, Indianapolis, IN), which were used as the dietary base. Food for the REF group contained 1% FO fat (RxOmega‐3 Factors, liquid, Natural Factors, Coquitlam, BC, Canada) and 6% beef fat (100% Pure beef dripping, Farmland, New Zealand) by weight, whereas those for the FO and SFA groups contained 12% FO and 3% beef fat, and 15% beef fat, respectively. The rats were housed three to a cage and fed their respective diets ad libitum for 6 weeks to 8 weeks. The body mass of each rat and the total food and water consumption per cage were monitored weekly throughout the feeding period.

### Preparation of the hearts

On an experimental day, a rat was delivered from the Animal Facility to the laboratory, under single‐blind protocol. It was deeply anesthetized with isoflurane (5% in O_2_) and killed by cervical dislocation. Thoracotomy and cardiectomy were performed and the heart was quickly placed in an ice‐cold saline bath to induce cardiac arrest. The aorta was rapidly cannulated and perfused to wash blood out of the coronary vasculature via Langendorff perfusion with oxygenated Tyrode solution, at a perfusion pressure of 70 mmHg, at room temperature. The Tyrode solution was composed of (mmol/L): 130 NaCl, 6 KCl, 1 MgCl_2_, 0.5 NaH_2_PO_4_, 1.5 CaCl_2_, 10 HEPES, and 10 glucose. The pH was adjusted to 7.4 using Tris. The solution was vigorously bubbled with 100% O_2_ throughout the experiment.

While being Langendorff perfused, and submerged under Tyrode solution, the large vessels were cannulated. One of the four pulmonary veins and the pulmonary artery were cannulated while the remaining vessels were ligated. Once cannulation was complete, perfusion was switched to prewarmed (37°C) Tyrode. A unipolar stimulus electrode (Coaxial Stimulation Electrode, Harvard Apparatus, MA) was placed on the right atrium to pace the heart at 5 Hz. The heart was placed inside a water‐jacketed chamber that maintained its temperature at 37°C and prevented surface desiccation.

Fiber‐optic oxygen sensors (FOXY‐R‐8CM, Ocean Optics Inc., Dunedin, FL) were placed in the solution just superior to the coronary ostia (“upstream”) and in the pulmonary artery cannula (“downstream”) to measure coronary arterial and venous partial pressure of oxygen (Po_2_), respectively. Two perivascular flow probes (T206 and T106, Transonic^®^ system, Ithaca, NY), modified for in “in‐line” configuration, were placed near the oxygen sensors to measure the rates of aortic and coronary flow. Pressure transducers (SP 844 Transducer, MEMSCAP, Crolles Cedex, France) were placed in the aortic outflow catheter, the left atrial cannula, and the pulmonary arterial cannula. Data acquisition and recording were achieved using PowerLab LabChart^®^ Pro software (ADInstruments, Dunedin, New Zealand).

### Working‐heart experiments

Once the heart had reached a steady‐state of coronary venous Po_2_, perfusion was switched from Langendorff mode to working‐heart mode (preload 10 mmHg, afterload 50 mmHg). By adjusting the height of the preload and afterload pressure heads, the LV filling pressure (preload) and afterload imposed on the heart could be changed independently. A range of preloads (5, 10, 15, and 20 mmHg) and afterloads (40, 60, 75, 85 and 95 mmHg) was adopted. Data were recorded continuously under each preload–afterload combination.

Left‐ventricular power, the rate of performing work (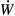, eq. 1), was calculated as the product of mean arterial pressure (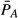), stroke volume (*V*_*s*_), and heart rate (5 Hz). Stroke volume was calculated as the quotient of arterial flow and heart rate. The rate of oxygen consumption (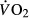, eq. 2) was given by the product of coronary flow rate (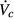), the difference between the upstream (*P*_*a*_) and downstream (*P*_*v*_) values of coronary Po_2_, and the solubility of oxygen in saline (*σ*) at 37°C (22.7 mLO_2_ atm^−1^ L^−1^). Total efficiency (*ε*_Total_, eq. 3) and mechanical efficiency (*ε*_Mech_, eq. 4) were calculated as the ratio of power and the appropriate rate of change of enthalpy. The rates of change of total enthalpy (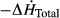) and basal enthalpy (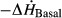) were calculated from their respective values of 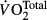, using the energetic equivalent of O_2_ (20 kJ/L). Following unsuccessful early attempts to use a commercial LV pressure–volume catheter, and reversion to the classical method of determining stroke volume, successful experiments were achieved with seven hearts from the REF group, five from the FO group, and seven from the SFA group.


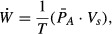
where *T* is the period between beats



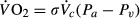





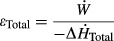





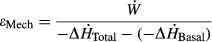



At the completion of an experiment, the atria were trimmed and discarded, the ventricles were blot dried and heart wet mass measured. A small sample of LV wall tissue was isolated and its wet mass measured. The sample was reweighed following drying in an oven at 60°C for 24 h. The wet weight:dry weight ratio of the sample was then applied to the whole heart so that its dry mass as well as the wet:dry weight ratio of the entire heart could be estimated. Experimental variables are expressed relative to the dry mass of the heart.

### Myocardial content of fatty acids

The remaining ventricular tissue was frozen in liquid N_2_ and stored at −80°C. From each of the three Diet groups, LV samples from three hearts were analyzed commercially (AsureQuality, Auckland, New Zealand) for myocardial fatty acid content.

### Statistical analyses

The data describing contractile power and efficiency as functions of afterload were fitted using third‐order polynomial functions and the peak values of the fitted functions determined using MATLAB^®^ software (Mathworks, Natick, MA). The average values for each variable of interest were tested for statistical significance by ANOVA (one‐way (“Diet”) for morphological data and three‐way (“Diet,” “preload” and “afterload”) for mechanoenergetic data), using the GLM (generalized linear model) facility of the SAS software package (SAS Institute Inc., Cary, IN). Results are presented as mean ± standard error (SEM). Statistical significance was declared at *p* < 0.05. Post hoc tests of differences among means were applied, when appropriate, using orthogonal contrast vectors.

## Results

### Effects of the diets on morphometric characteristics of the rats and hearts

**Figure 1.**
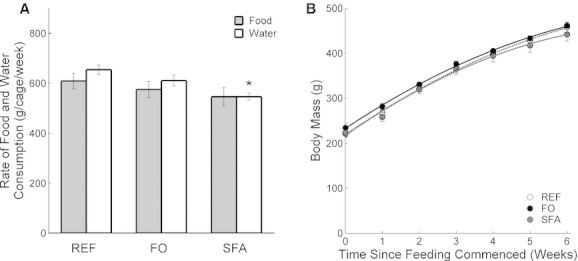
(A) Average rate of food and water consumption by three rats housed in the same cage, and (B) average body mass of the rats over the feeding period (6 weeks) for the three Diet groups (*n *=**12 each group). Values are mean ± SEM. Significantly less water consumption in the SFA group with respect to both REF and FO groups (A).

The rates of food and water consumption, and the average body mass of the rats in each Diet group over the 6‐week feeding period, are presented in Figure 1. There was no effect of diet on the rate of food consumption, whereas the SFA group consumed less water (Fig. 1A). The average body mass for each Diet group is shown in Figure 1B. There was no difference in the body mass among the Diet groups throughout the dietary period.

The average mass of the animals and their hearts used in the experiments is summarized in Table 1. The average final body mass of the rats at the time of experiment was the same across the Diet groups. ANOVA revealed a difference among the Diet groups in the blotted wet weights of the hearts; post hoc analysis confirmed that the SFA hearts were lighter than those of the other two Diet groups. However, there were no differences in the heart wet‐weight:body‐weight ratios, heart dry mass, or the ratio of wet to dry mass of the hearts among the Diet groups.

**Table 1.** Mass of rats and hearts

	REF (*n *=**9)	FO (*n *=**7)	SFA (*n *=**9)
	Mean ± SEM		
Body mass (g)	479.6 ± 8.9	475.3 ± 7.2	451.9 ± 18.4
Heart wet mass (g)	1.50 ± 0.06	1.53 ± 0.04	1.30* ± 0.03
Heart wet/Body (%)	0.31 ± 0.01	0.32 ± 0.01	0.29 ± 0.01
Heart dry mass (g)	0.26 ± 0.01	0.27 ± 0.01	0.24 ± 0.01
Heart wet/dry mass	5.79 ± 0.15	5.73 ± 0.12	5.53 ± 0.13

Values are mean ± SEM for *n* observations; *P < 0.05.

### Fatty acid contents of diets and hearts

Table 2 summarizes the fatty acid contents of the rat chows and hearts in the current study (*n* = 3 per group). The level of EPA and DHA found in the foods and hearts was considerably higher in the FO group compared to the other two Diet groups. For ease of comparison, we have duplicated the equivalent measurements from Table 1 of Pepe and McLennan (1996). Whereas there are minor quantitative differences, both the dietary components and myocardial results are qualitatively similar between our studies.

**Table 2.** Summary of fatty acid analyses of the prepared rat chows (“Dietary”) and the hearts (“Myocardial”)

	Current Study						Pepe & McLennan (1996)					
	Dietary			Myocardial			Dietary			Myocardial		
	REF	FO	SFA	REF	FO	SFA	REF	FO	SFA	REF	FO	SFA
	mol/100 mol											
18:2 (n‐6), LNA	1.1	0.9	0.8	5.8	1.7	8.0	33.9	5.6	6.7	21.6	10.2	13.9
18:3 (n‐3), ALNA	0.7	0.5	0.7	0.2	<0.1	0.4	3.3	1.2	1.5	0.1	0.1	0.1
20:4 (n‐6), AA	0.3	1.5	<0.1	13.6	15.6	17.3	0.6	1.0	0.1	17.7	14.1	20.3
20:5 (n‐3), EPA	4.9	26.8	<0.1	2.3	3.9	0.2	2.2	24.3	0.4	0.3	3.1	0.3
22:5 (n‐3), DPA	0.7	3.7	<0.1	3.5	3.4	1.2	‐	1.5	0.1	1.4	1.8	1.8
22:6 (n‐3), DHA	2.4	13.4	<0.1	15.1	21.2	9.1	6.2	11.8	0.8	15.4	27.2	19.5
Σ (n‐6) PUFA	2.6	4.8	1.5	19.8	16.7	27	34.5	6.6	7.0	39.8	24.7	34.6
Σ (n‐3) PUFA	9.7	46.4	1.5	21.7	28.9	11.2	11.8	38.9	2.8	17.2	32.3	21.4
(n‐3):(n‐6 PUFA)	3.7	9.6	1.0	1.1	1.8	0.4	0.3	5.9	0.4	0.4	1.3	0.6
Σ SFA	50.5	23.8	55.7	34.2	35.3	36.1	25.3	25.3	54.9	33.9	34.9	34.8
Σ MUFA	33.3	21.9	34.9	15.4	11.1	17.8	–	–	–	–	–	–
Σ PUFA	12.8	51.5	3.5	41.7	45.7	38.4	46.2	45.5	9.6	57.0	57.0	56.0
PUFA:SFA	0.3	2.2	0.1	1.2	1.3	1.1	1.6	1.8	0.2	1.7	1.6	1.7
PUFA:MUFA	0.4	2.4	0.1	2.7	4.1	2.2	–	–	–	–	–	–
Total fat content (% by weight)	4.1	8.5	8.8	1.7	1.8	1.9	7.6	15.3	15.3	–	–	–

### Isolated working‐heart experiments

**Figure 2.**
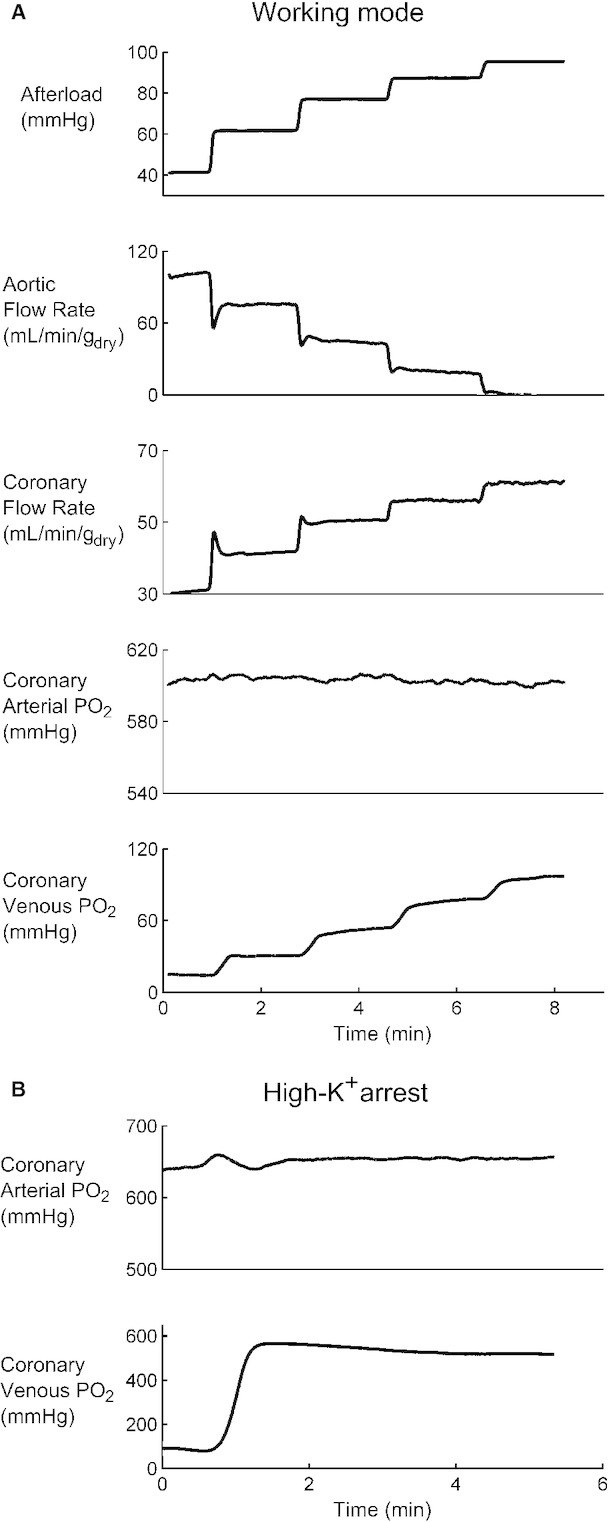
Typical experimental data traces, post processed following acquisition and recording by the LabChart Pro^®^ interface during a working‐heart experiment. Instantaneous measurements of perfusate flows and upstream and downstream PO_2_ are shown for working‐heart interventions at various afterloads: (40, 60, 75, 85, and 95) mmHg (A), and before and during high‐K^+^ arrest (B).

A total of seven different mechanoenergetic indices were simultaneously monitored and recorded, selected examples of which are presented in Figure 2. Distinctive changes, particularly in the aortic and coronary outflow rates and downstream (coronary venous) Po_2_, are evident in response to stepwise increases in afterload (Fig. 2A). A dramatic increase in the downstream Po_2_ is also evident following administration of a cardioplegic high‐K^+^ solution (Fig. 2B).

### Aortic and coronary flow rates as functions of afterload and preload

**Figure 3.**
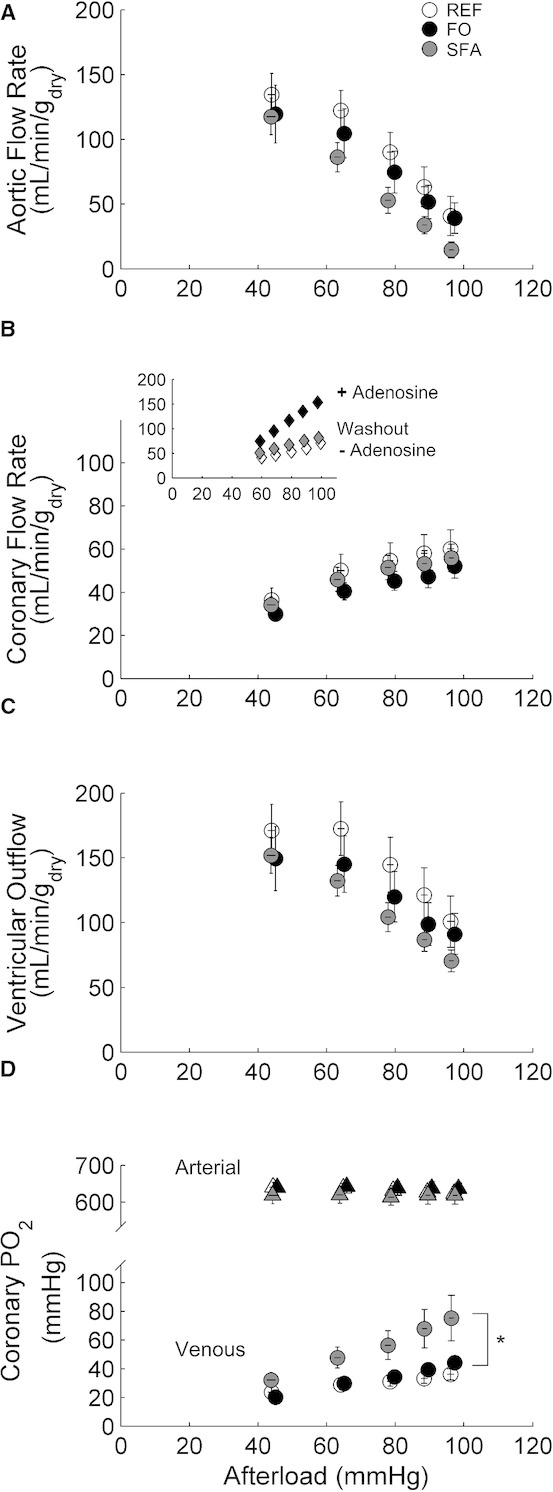
Aortic (A) and coronary (B) flow rates and total ventricular outflow (C), as functions of afterload at preload 10 mmHg. (D) Coronary arterial and venous PO_2_ (note the two different scales on the ordinate); significant elevation in coronary venous PO_2_ at all afterloads in the SFA group. Symbols represent mean ± SEM of *n* = 7 REF (open symbols), *n *=**5 FO (black symbols), and *n *=**7 SFA (gray symbols) Diet groups.

Figure 3 shows that there was no difference in aortic (Panel A) or coronary (Panel B) flow rates, or their sum (total ventricular outflow, Panel**C) among the Diet groups. Whereas arterial Po_2_ remained constant, coronary venous Po_2_ progressively increased with afterload, and was greater at all afterloads in the SFA group (Panel D). The inset in Panel B shows the effect of 2 *μ*M adenosine on coronary flow rate over a range of afterloads. Addition of adenosine significantly increased the coronary flow rate, which was reversed by approximately 5 min of washout with adenosine‐free perfusate. Comparable results were obtained in a second heart (data not shown).

### Mechanoenergetic variables as functions of afterload and preload

**Figure 4.**
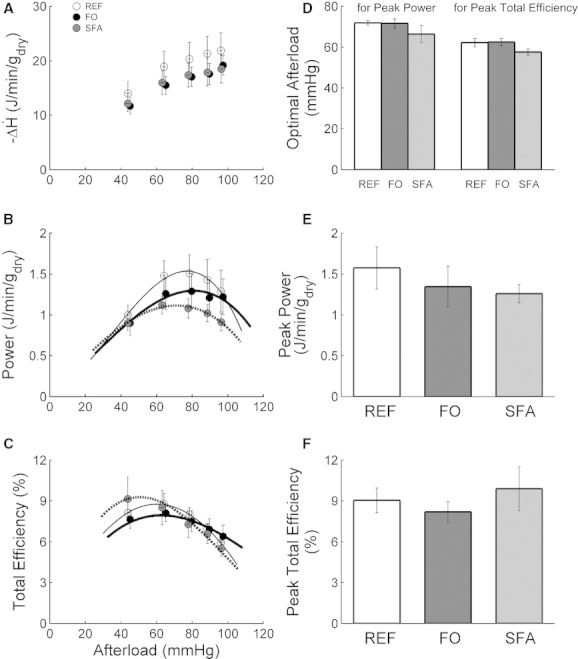
Power (B), change of enthalpy (A) and their ratio, total efficiency (C), as functions of afterload at a preload of 10 mmHg, for the three Diet groups. Power and efficiency data fitted with third‐order polynomial functions. Means ± SEMs of *n* = 7 REF (open circles), *n* = 5 FO (filled circles), and *n* = 7 SFA (gray circles) working hearts. Peak values of power (E) and total efficiency (F), and the afterloads at which they occurred (D).

Figure 4 reveals average changes in enthalpy (A), power (B), and total efficiency (C), as functions of afterload, at a preload of 10 mmHg. The numeric values at the peaks of the power (E) and total efficiency (F) relations for each dietary group are shown in E and F, respectively. The afterloads at which the peak values of both variables occurred are presented in D. Comparable measurements were made at each of the other three preloads, but, to simplify presentation, we show the effect of afterload at only a single preload (10 mmHg). ANOVAs revealed no differences in peak values or optimal afterloads among the Diet groups under any combination of preload and afterload.

**Figure 5.**
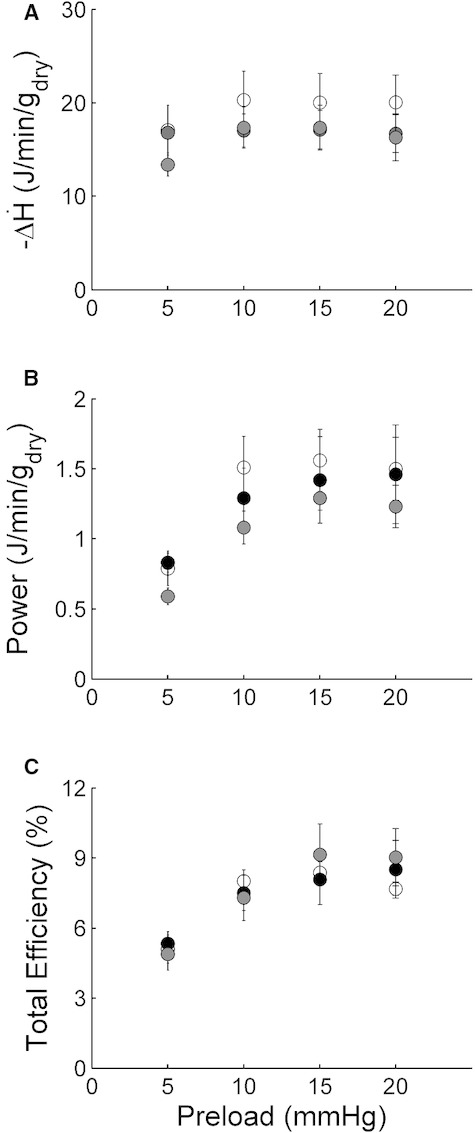
Change of enthalpy (A), power (B), and total efficiency (C) as functions of preload at afterload 75 mmHg. No difference among Diet groups for any variable; mean ± SEM from *n *=**7 REF,* n *=**5 FO, and *n* = 7 SFA hearts.

The results shown in Figure 5 are complementary to those of Figure 4. They show change of enthalpy (A), power (B) and their ratio, and total efficiency (C), as functions of preload, at afterload 75 mmHg. Once again, there was no difference among the three Diet groups across any of the three variables.

### Basal oxygen consumption and mechanical efficiency

**Figure 6.**
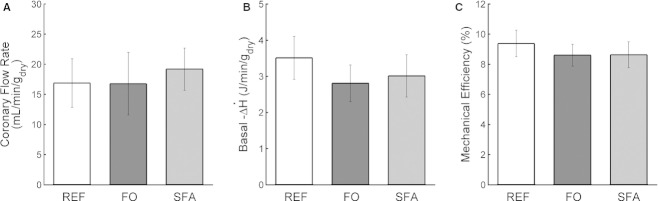
Coronary flow rate (A) and change of enthalpy (B) during high‐K^+^ arrest. (C) Average mechanical efficiencies of the three Diet groups at preload 10 mmHg and afterload 75 mmHg. Bars denote mean ± SEM from *n* = 7 REF,* n *=**5 FO, and *n* = 7 SFA hearts. No statistically significant differences among Diet groups.

Basal metabolic rate was indexed as the steady‐state rate of oxygen consumption during high‐K^+^ (26 mmol/L) cardiac arrest (Fig. 2B). Importantly, there were no differences in average coronary flow rates among the Diet groups during the period of arrest (Fig. 6A), although the rates were much lower than when the hearts were working (*cf* Fig. 3B). Oxygen consumption was converted to change of enthalpy, and likewise revealed no dependency on diet (Fig. 6B). Subtraction of basal enthalpy from total enthalpy, in the denominator of equation 3, yields the expression for (suprabasal) mechanical efficiency (eq. 4). Average mechanical efficiency was calculated for each Diet group and the values were presented in Figure 6C for preload 10 mmHg and afterload 75 mmHg. As there were no variations in either total enthalpy (Fig. 4C and F) or basal enthalpy (Fig. 6) among the Diet groups, there were likewise no differences in mechanical efficiency: 9.4 ± 0.9%, 8.6 ± 0.7%, and 8.6 ± 0.9% for the REF, FO, and SFA groups, respectively.

## Discussion

In this study, we have investigated the consequences to the mechanoenergetic performance of the isolated hearts of rats fed one of three diets: reference (REF), fish oil‐supplemented (FO), or saturated fatty acid‐supplemented (SFA) food. The constituents of the diets were chosen to duplicate, as closely as possible, those used by Pepe and McLennan (2002, 2007) in their studies showing dramatic effects of diet on cardiac efficiency. The 6‐ to 8‐week dietary intervention was successful in achieving pronounced differences in unsaturated and saturated fatty acids in the myocardium (Table 2), thereby justifying comparison of our results with those from comparable studies reported in the literature.

The most comprehensive treatments of the effects of both saturated and unsaturated fats, of which we are aware, are those of Pepe & McLennan (2002, 2007), who found the lowest cardiac efficiency (3%) in isolated hearts of rats fed saturated animal fats, the highest (10%) in rats on a fish oil diet, and an intermediate value (5–6%) in hearts of animals fed a reference diet. As our null results (Figs. 3–6), which show a diet‐indifferent value of total efficiency (9–10%), are at variance with theirs, it is critical that we compare our respective methodologies. The obvious departure between our two studies is our use of a crystalloid perfusate (Tyrode solution) and their use of an erythrocyte‐enhanced (40% hematocrit) medium. Although there was a negligible difference in arterial Po_2_ between the two studies, the difference in oxygen content was considerable. Does that matter to the outcome? We consider that it matters much less than is sometimes claimed, primarily because it is the partial pressure of oxygen, rather than its content, that drives its diffusion out of the coronary capillaries to the respiring mitochondria. Several experimental tests of adequacy of oxygenation obtained. First, we observed (Fig. 3D) that venous Po_2_ increased with afterload, despite the increase in energy demand. It is difficult to believe that oxygenation was inadequate at some lower workload when it *increased* at a higher workload. Second, we note that the lowest value of venous Po_2_, 20–30 mmHg (Fig. 3D), is comparable to that of whole‐body, mixed‐venous Po_2_ under conditions of moderate‐to‐heavy exercise. Third, the data of Figure 3B (inset) convincingly reveal the presence of coronary flow reserve at all afterloads. Had the heart been starved of oxygen, at any afterload, it certainly had the inherent ability to counter starvation by vasodilation.

A second departure from the protocol adopted by Pepe and McLennan (2002) was our use of HEPES‐buffered Tyrode solution instead of bicarbonate‐buffered Krebs–Henseleit. Whereas the use of Tyrode perfusate provided a 5% increase in oxygen fraction (95% to 100%), thereby slightly increasing both the content of oxygen and its diffusing capacity, it too is a crystalloid medium with inevitable consequences for tissue edema, as shown by the elevated wet:dry ratios of heart mass in all three dietary groups (Table 1). Tissue edema increases the diffusion distance for oxygen, thereby countering the advantage of higher Po_2_. Curiously, our average wet:dry ratio of 5.7 for Tyrode‐perfused hearts is lower than the value of 6.04 reported by Pepe and McLennan (1996) for Krebs–Henseleit‐perfused hearts.

But our principal justification for adopting a HEPES‐buffered, instead of a bicarbonate‐buffered, perfusate arises from the results of mathematical modeling. Simulations, using a full model of the known ionic exchangers of the cardiac cell (Crampin and Smith 2006), including mitochondrial dependence on metabolites, reveal that abruptly switching from HEPES to bicarbonate causes a transient decrease in force production, whereas the converse switch causes a transient increase, the *steady‐state* levels being identical in both cases. These transient changes in force reflect the time taken to reestablish intracellular pH following a change in extracellular CO_2_ (Goo et al. 2011). As simulated steady‐state force is unaffected, we predict an absence of effect on cell shortening and, hence, no effect on either work or efficiency.

A third point of departure is the difference in duration of the dietary periods (6 weeks vs. 16 weeks) and, hence, the difference in age of the animals at testing (14 weeks vs. 22 weeks). It seems unlikely to us that either difference could make a substantive contribution to the observed diet‐dependent differences in efficiency. It is known that the incorporation of FO fatty acids into cellular membranes is rapid, achieving saturation within 3 weeks of feeding (Tahin et al. 1981). Furthermore, the difference in age of the rats at the time of experimentation is also unlikely to have contributed, as the decline in myocardial efficiency, even in rats as old as 2 years, is modest (Starnes and Rumsey 1988).

Having considered several differences in technique between our studies, we emphasize three similarities. The most important of these is the fact that the dietary regimes were comparable between the two studies. As shown in Table 2, the concentrations of saturated fats in the myocardial tissues of SFA‐fed animals and the complementary concentrations of unsaturated fats in the FO group give confidence that the hearts had responded similarly to their respective diets in both studies. Second, and of unknown relevance, we mimicked the prior investigations by using male rats of the Wistar strain. Third, both studies were conducted using isolated hearts maintained at body temperature (37°C).

All similarities and differences in methodologies aside, we find the striking difference in cardioenergetic results inexplicable. Our sole point of agreement regards work output. In both their 2002 and 2007 studies, Pepe and McLennan found comparable work rates across all three groups (albeit at the single afterload of 75 mmHg). Our results at 10 mmHg preload and variable afterload (Fig. 4), at 75 mmHg afterload and variable preload (Fig. 5), and at all other preload–afterload combinations (data not shown) are in accord. However, although Pepe and McLennan (2002, 2007) found dramatic differences in oxygen consumption and, in consequence, cardiac efficiency, among their dietary groups, we found none (Figs. 4A,C and F, 5A and C, and 6C). A possible (albeit unlikely) explanation is that their selection of 75 mmHg afterload either may not have been optimal or, alternatively, the optimum may have been diet dependent. Our use of a range of afterloads, whereby unambiguous peak values of work (or power) and efficiency were revealed (Fig. 4D and F), has obviated this potential problem.

There is, however, an additional point of difference that may be relevant: the pronounced diet‐dependent differences in rates of coronary flow in the studies by Pepe and McLennan (2002, 2007). In their 2007 publication, an increase in the dietary FO, from 0% (12% saturated fats) to 12% (with unreported consequences for myocardial concentrations), was accompanied by a dose‐dependent, eightfold increase in efficiency, and a converse fivefold, dose‐dependent decrease in the rate of O_2_ consumption. The decrease in O_2_ consumption, in turn, was associated with a threefold decrease in coronary flow rate. A comparable effect of coronary flow rate on the rate of O_2_ consumption, thus on cardiac efficiency, was also apparent in their 2002 publication. Such a striking variation in coronary flow, at constant preload (10 mmHg) and constant afterload (75 mmHg) is baffling, although a more modest, dietary fat‐dependent increase in coronary flow has been observed by Cole et al. (2011). In contrast, we saw no effect of diet, under the identical conditions of preload and afterload, in our experiments (Fig. 3A).

Because we are unable to conceive of the mitochondrial changes in P:O ratios that would be required to underwrite an eightfold difference in cardiac efficiency in response to a difference in diet from 12% saturated fats to 12% fish oils (figure 1C of Pepe & McLennan, 2007), we suspect the involvement of a hemodynamic effect. In this regard, it is interesting that Pagliaro et al. (2002) found that fatty acid metabolism is required in order to elicit Gregg's phenomenon (Gregg 1963) – that is, coronary flow dependence of the rate of cardiac oxygen consumption. However, as coronary flow was varied only between, but not within, a given dietary dose of fish oil, little more can be inferred.

Finally, we address two theoretical concerns: the potential effects of substrate and the energetic equivalence of oxygen on the calculation of efficiency. We reemphasize that our use of glucose as exogenous substrate was required in order to mimic the protocols adopted by Pepe and McLennan (2002, 2007). Nevertheless, the amount of ATP produced per mole of O_2_ is metabolite dependent. For example, oxidation of 1 mole of glucose requires 6 moles of O_2_, yielding 38 moles of ATP, providing the ATP/O_2_ ratio of 6.3 and RQ of 1. If, instead, 1 mole of palmitate, a 16‐carbon SFA, is oxidized, it generates 129 moles of ATP, producing the ATP/O_2_ ratio of 5.6 and RQ of 0.7. These stoichiometric values, giving an “average” P:O ratio of approximately 3, have appeared in generations of textbooks. However, recent investigations suggest the number of ATP molecules produced from oxidation of glucose and palmitate to be 31 and 104, respectively (Salway 2004). These lower values of the stoichiometric constants would reduce the P:O ratio to roughly 2.5, thereby increasing estimates of cardiac efficiency by approximately 20% (e.g., from 10% to 12%). Note that this 13.3% difference in yield of ATP represents an extreme estimate – based on metabolizing either pure glucose (RQ = 1) or pure palmitate (RQ = 0.7). However, regardless of its precise value, the P:O ratio can have no *qualitative* influence on the differential effects of diet on cardiac efficiency. Likewise, whereas the numeric value for the energetic equivalent of oxygen affects the numeric value of efficiency, it can have no influence on differences in the latter among the dietary groups.

Hence, we arrive at an impasse. Pepe and McLennan (2002) present clear evidence that, in the isolated rat heart and with respect to a normal diet, dietary supplementation with polyunsaturated fatty acids increases cardiac efficiency while a diet high in saturated fats reduces efficiency. Furthermore, they show the efficiency effect of fish oil to be dietary dose dependent (Pepe and McLennan 2007). We, in contrast, found no effect on cardiac efficiency of dietary supplementation with either saturated or unsaturated fatty acids. The latter finding is in accord with that of others in comparable studies of isolated adult rat hearts (de Deckere and ten Hoor 1979; De Deckere and Ten Hoor 1980; Demaison et al. 2000) and juvenile pig hearts (Hartog et al. 1987) in which various combinations of mackerel oil, sunflower seed oil, hydrogenated coconut oil, and lard were compared. We conclude that, whereas the composition of dietary fatty acids has demonstrable consequences for cardiovascular health, their *modus operandi* is not via an effect on the contractile efficiency of the heart.

## Conflict of Interest

None of the authors has any conflict of interest to disclose.
